# Functional Coupling of Human Microphysiology Systems: Intestine, Liver, Kidney Proximal Tubule, Blood-Brain Barrier and Skeletal Muscle

**DOI:** 10.1038/srep42296

**Published:** 2017-02-08

**Authors:** Lawrence Vernetti, Albert Gough, Nicholas Baetz, Sarah Blutt, James R. Broughman, Jacquelyn A. Brown, Jennifer Foulke-Abel, Nesrin Hasan, Julie In, Edward Kelly, Olga Kovbasnjuk, Jonathan Repper, Nina Senutovitch, Janet Stabb, Catherine Yeung, Nick C. Zachos, Mark Donowitz, Mary Estes, Jonathan Himmelfarb, George Truskey, John P. Wikswo, D. Lansing Taylor

**Affiliations:** 1 University of Pittsburgh, Drug Discovery Institute Pittsburgh, PA, USA; 2 Department of Computational and Systems Biology, University of Pittsburgh, Baltimore, PA, USA; 3 Departments of Physiology and Medicine, GI Division, Johns Hopkins University School of Medicine, Baltimore, Maryland, USA; 4 Departments of Molecular Virology and Microbiology and Medicine, Baylor College of Medicine, Houston, TX, USA; 5 Department of Physics and Astronomy, Vanderbilt Institute for Integrative Biosystems Research and Education, Vanderbilt University, Nashville, TN, USA; 6 Department of Pharmaceutics, University of Washington, WA, USA; 7 Department of Biomedical Engineering, Duke University, Durham, NC, USA; 8 Department of Pharmacy, University of Washington, WA, USA; 9 Kidney Research Institute, University of Washington, WA, USA; 10 Department of Medicine, University of Washington, WA, USA; 11 Department of Biomedical Engineering, Vanderbilt University, Nashville, TN, USA; 12 University of Pittsburgh Cancer Institute, PA, USA.

## Abstract

Organ interactions resulting from drug, metabolite or xenobiotic transport between organs are key components of human metabolism that impact therapeutic action and toxic side effects. Preclinical animal testing often fails to predict adverse outcomes arising from sequential, multi-organ metabolism of drugs and xenobiotics. Human microphysiological systems (MPS) can model these interactions and are predicted to dramatically improve the efficiency of the drug development process. In this study, five human MPS models were evaluated for functional coupling, defined as the determination of organ interactions via an *in vivo-*like sequential, organ-to-organ transfer of media. MPS models representing the major absorption, metabolism and clearance organs (the jejunum, liver and kidney) were evaluated, along with skeletal muscle and neurovascular models. Three compounds were evaluated for organ-specific processing: terfenadine for pharmacokinetics (PK) and toxicity; trimethylamine (TMA) as a potentially toxic microbiome metabolite; and vitamin D3. We show that the organ-specific processing of these compounds was consistent with clinical data, and discovered that trimethylamine-N-oxide (TMAO) crosses the blood-brain barrier. These studies demonstrate the potential of human MPS for multi-organ toxicity and absorption, distribution, metabolism and excretion (ADME), provide guidance for physically coupling MPS, and offer an approach to coupling MPS with distinct media and perfusion requirements.

The goal of *in vitro* and *in vivo* toxicity testing is to identify compounds that would predict adverse reactions in humans. Olson *et al*.^1^ found that only 70% of human toxicity was predicted from animal testing. Currently we rely on traditional toxicity testing in animals, a 1930’s methodology that is now challenged due to questionable relevance to human risk, high cost, ethical concerns, and throughput that is too limited for the nearly 80,000 industrial chemicals not yet tested for safety. Additionally, testing usually extrapolates acute, high dose animal results to chronic, low dose human exposures, thereby risking rejection or limiting the use of drugs, industrial chemicals or consumer products. Moreover, the ability of lab animal target organ toxicity to predict dose-limiting toxicity in the corresponding human organ varies widely, from a low of 30% for human cutaneous toxicity, to 50–60% for human hepatotoxicity, to a high of 90% for hematological drug toxicity^1^. Animal drug efficacy models are also notoriously discordant. In an analysis of six drugs to treat head injury, hemorrhage, acute ischemic stroke, neonatal respiratory distress syndrome, and osteoporosis, it was found that efficacy was similar in animals and humans for three drugs but was dissimilar for another three^2^. In oncology drug development, animal models often over-predict anti-tumor efficacy in humans^3^,^4^. Examples such as these highlight the need to continue research into methods that reduce the dependence on laboratory animals for toxicity testing of environmental chemicals, determine efficacy and toxicity in drug development, serve as a mimic of human diseases, and provide patient-specific guidance in the emerging field of precision medicine.

Recent advances in bioengineered materials, microfluidic technology, and the availability of human primary, immortalized, and induced pluripotent stem cell (iPSC)-derived cells are enabling development of human microphysiological systems (MPS), sometimes called “organs-on-a-chip” or “human-on-a-chip,” that use multiple organ-specific human cells to recapitulate many functional and structural properties of a human organ. It is now generally accepted and supported by data that cellular responses to drugs in most human organs are more accurately approximated in 3D cell cultures than in traditional static 2D cell cultures^5^,^6^. Microfluidic perfusion further improves model performance by providing a flow of nutrients and oxygen and the removal of waste products from the cell cultures^7^. Physiologically relevant flow increases oxygen consumption, Krebs cycle activity and secretion of synthesized proteins, and decreases expression of the hypoxia HIF1 gene. Flow also improves the absorption and metabolism of compounds like benzo[a]pyrene^6^,^8^,^9^. The large number of recent publications reviewing organ MPS models indicates a high degree of interest by industrial and academic researchers, granting agencies and other stakeholders^10^,^11^,^12^,^13^. In addition to the stand-alone MPS, investigators are linking MPS to study organ-organ functional interactions, efficacy, PK and toxicology^14^,^15^,^16^,^17^,^18^.

An obvious approach to linking organs is direct coupling of the media stream outflow from one organ into the inflow of the next by use of tubing or a connecting channel. Some limitations to this approach include the requirement for a common medium, difficulty in reducing metabolic wastes to the next organ, organ-specific flow rates and adequate oxygenation of all modules in the system^19^. These requirements are most easily addressed when the linked organ modules are designed and developed at the same time and in the same laboratory, but even when the organ modules are co-developed, the proper scaling between organ modules is a significant design and calculation challenge. Although organ modules can be sized using allometric scaling^20^, the resulting functional capacity of the individual organ models may not scale the same. An alternative to direct or “physical” coupling is functional coupling, which we define as the transfer of media from one organ module to the next in a physiological sequence, wherein each organ module functionally transforms the media composition based upon the specific metabolic activity of that module.

Some advantages of functional coupling include the ability to adjust flow rates and media composition for each module, relaxing the requirement that all modules must be set up and operated at the same location and/or time, and the possibility that the functional performance of each module can be evaluated before media is transferred to the next module. As a preliminary step to solving the challenges of direct coupling of MPS organs in one continuous fluid stream, functional coupling provides important information about the optimal functional scaling of each organ module in a coupled system, and any issues of media compatibility between modules.

In this report, we present the results from functional coupling of four human MPS models: the human intestine (Johns Hopkins University [JHU]/Baylor College of Medicine [Baylor]), the sequentially layered self-assembly liver (SQL-SAL, University of Pittsburgh [UPitt]), a vascularized or non-vascularized proximal tubule kidney (VPTK or PTK, University of Washington [UWash]), and an intact blood-brain barrier/neurovascular unit (BBB/NVU, Vanderbilt University). The intestine, liver and kidney represent the major ADME organs involved in uptake, metabolism and elimination of most orally absorbed small molecules and are thus three important organs for human PK. The blood-brain barrier was included as it is often a confounding tissue barrier for the pharmaceutical industry. Terfenadine, trimethylamine and vitamin D3 were investigated by functional coupling in these organ modules, and we tracked their absorption, metabolism and excretion. We also present the results from terfenadine exposure in functionally coupled static liver and skeletal muscle myobundle (muscle) modules (Duke). Coupling the human liver and muscle modules was a test of metabolism-based toxicity on a potential “off target” organ. Terfenadine was chosen for its known transport and metabolism in the intestine and liver, its potential as a cardiac toxin, and its inhibitory effect on the skeletal muscle eag2 potassium channel^21^,^22^. Trimethylamine is a microbiome product that can be metabolized in the liver to a potential renal toxin^23^. Finally, an oral administration model of vitamin D3 was chosen to evaluate the liver and kidney metabolic conversion to 1α, 25 (OH)_2_ vitamin D3 and to evaluate transport of vitamin D3 and its metabolites across the BBB.

## Results

**Figure 1:**
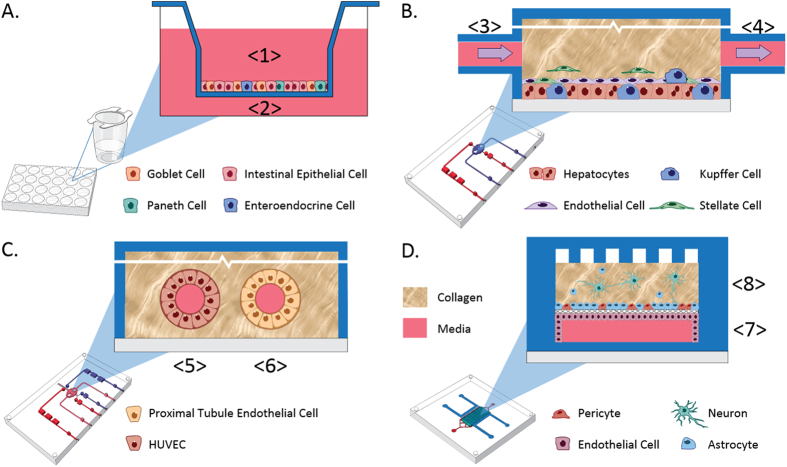
Schematic representations of the four of the organ systems used for functional coupling. (**A**) The intestinal module is constructed in transwells from primary jejunum enteroids. Test agents are applied in the apical compartment <1>. The media collected in the basolateral compartment <2> is used to add to the liver. (**B**) Media from the jejunum intestine basolateral compartment <2> is perfused as a 1:3 jejunum/naïve liver media into the influx port of the SQL-SAL liver model <3>. Efflux media is collected <4> and used to add to two downstream organ models. (**C**) The vascularized kidney proximal tubule module is a two lumen, dual perfusion system. For the vascular compartment, jejunum/liver-conditioned media <4> is diluted 1:2 or 1:4 with naïve EGM-2 media and then perfused into the influx port <5> to collect effluent from the proximal tubule at <6>. In parallel with perfusion through the vascular compartment, the proximal tubule compartment is perfused with naïve DMEM/F12 PTEC media <6> for effluent collection. (**D**) The blood-brain barrier with NVU is constructed in a membrane-separated, two-chambered microfluidic device. The brain-derived endothelial vascular compartment is perfused at the influx port <7> with jejunum/liver-conditioned media <4> diluted 1:4 with naïve EGM-2 media. The effluent is collected at the efflux port <7>. In parallel with perfusion through the vascular compartment, the neuronal cell compartment is perfused with naïve EBM-2 media at the influx port <8> for effluent collection at <8>.

**Figure 2:**
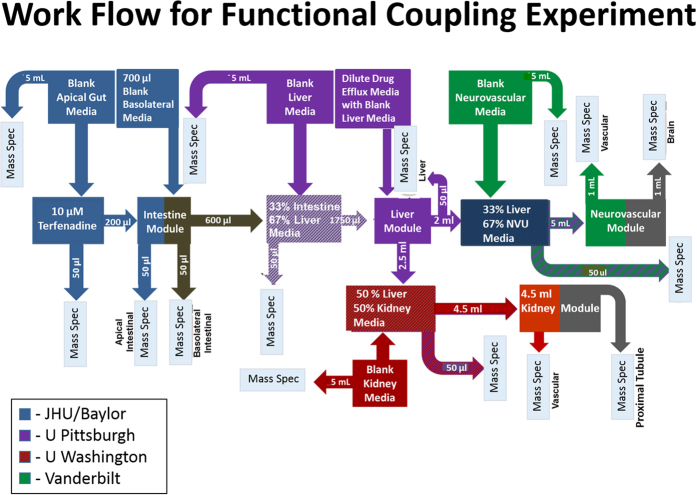
Work flow for functional coupling experiments. Terfenadine exposure is used as an example; TMA and vitamin D3 experiments followed essentially the same workflow. The test compound is initially added to the apical gut media and samples are collected from the apical and basolateral media for MS analysis. Basolateral media samples are sent to UPitt where they are mixed with liver media for exposure in the liver module. Effluent samples are taken for MS analysis and sent to UWash and Vanderbilt where they are mixed with kidney and NVU media, respectively. Samples are taken of the effluent from the kidney proximal tubule module and from the vascular and brain sides of the NVU for MS analysis.

These results are from the collaborative effort of six universities to evaluate the transport and metabolism of three test agents in four independently developed and functionally coupled multicellular MPS organ models as described in Table 1. A second experiment evaluated terfenadine toxicity in a human liver model functionally coupled to a muscle model. In both experiments the models were linked through functional coupling, a method which allows each organ to be operated independently under optimal perfusion conditions. Figure 1 illustrates the architectures of the four MPS models, and Fig. 2 illustrates the work flow for the processing of media containing terfenadine, from the intestine to the liver and finally to the kidney and BBB. All data were measured as concentrations by mass spectrometry (MS) using the protocols summarized in Supplemental Table S1.

### Four-organ functional coupling for analysis of terfenadine

**Figure 3:**
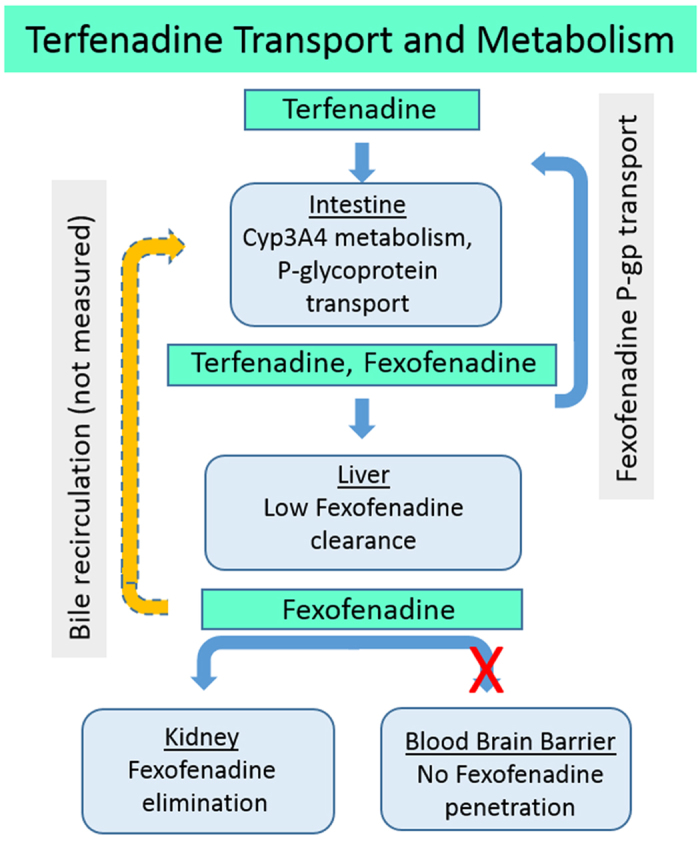
Functional analysis of terfenadine transport and metabolism in human organs. Terfenadine taken orally is absorbed and metabolized to fexofenadine in the intestine. Counter-transport carries fexofenadine back to the apical side of the intestinal wall. Remaining terfenadine is metabolized to fexofenadine in the liver. Fexofenadine is not able to cross the blood-brain barrier and is excreted by the kidney.

Figure 3 summarizes the *in vivo* uptake and metabolism of terfenadine and the transport and clearance of its CYP 3A4 metabolite fexofenadine. Terfenadine (10 μM) was added to the apical compartment of differentiated jejunal enteroids. At 24 h, 389 ± 62 ng/mL (88%) and 54 ± 28 ng/mL (12%) of the recoverable terfenadine was found in the apical and basolateral compartments, respectively (Supplemental Table S2). Fexofenadine concentrations in the apical and basolateral compartments were 44.4 ± 7.3 and 26.4 ± 3.5 ng/mL, respectively. These results demonstrated: a) functional CYP3A4 metabolism in the human intestine module; b) polarized transport of terfenadine from apical to basolateral; and c) transport of fexofenadine into the apical and basolateral compartments. These findings are consistent with the transport and metabolism of terfenadine and the metabolite fexofenadine in the human intestinal mucosa. The basolateral media was transferred to UPitt, where it was diluted 1: 3 in hepatocyte maintenance media for perfusion through the liver model (Fig. 2). After 24 h, terfenadine levels were 0.1 ng/mL in the efflux media while fexofenadine levels were 21.4 ng/mL. The low terfenadine level is consistent with clinical bioavailability at <1%. Also evident in clinical studies, greater than 95% of fexofenadine passes unchanged through the liver. Assuming all the fexofenadine passed through the liver model unchanged, at least 65% of the terfenadine was converted to fexofenadine and found in the efflux media. The liver-conditioned media was sent to Vanderbilt and UWash (Fig. 2). In the BBB/NVU, after 24 h, fexofenadine was found only in the vascular efflux (4.1 ± 0.3 ng/mL) and was below the level of detection in the neuronal efflux. In a follow-up study, with terfenadine in vascular media at 30 ng/mL and fexofenadine at 50 ng/mL, the findings confirmed that fexofenadine fails to cross the BBB, consistent with results in humans^24^. At UWash, the liver-conditioned media was perfused through the vascularized kidney model for 6 h. The majority of the fexofenadine (10.2 ± 3.6 ng/mL) was found in the vascular efflux media, but a small amount (0.1 ± 0.1 ng/mL), approximately equal to 1% of the total, was found in the lumen media (Supplemental Table S2).

### Two-organ liver-muscle coupling for terfenadine toxicity testing

**Figure 4:**
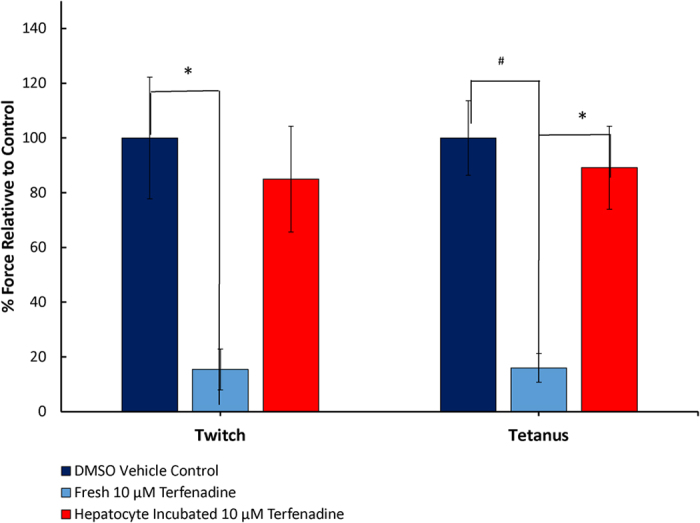
Effect of functional integration on terfenadine toxicity. Pre-conditioned terfenadine in the static liver model results in decreased effect on skeletal myobundles. Results are for one donor, N = 3 and presented as Mean ± S.D. *p < 0.05, ^#^*p* < 0.01.

Hepatocytes in the collagen sandwich assay format in 24-well plates were exposed to 10 μM terfenadine for 96 h, then the medium was transferred to the skeletal muscle myobundle module^25^. In myobundles treated for 24 h with medium containing 10 μM terfenadine there was a significant reduction in the twitch and tetanus force (Fig. 4); however, medium containing 10 μM terfenadine that was pre-conditioned by hepatocytes for 96 h did not reduce the twitch and tetanus force below control levels (Fig. 3). This suggests that the metabolism of terfenadine by the liver model was sufficient to reduce the toxicity to near control levels.

### Four-organ coupling for trimethylamine processing

**Figure 5:**
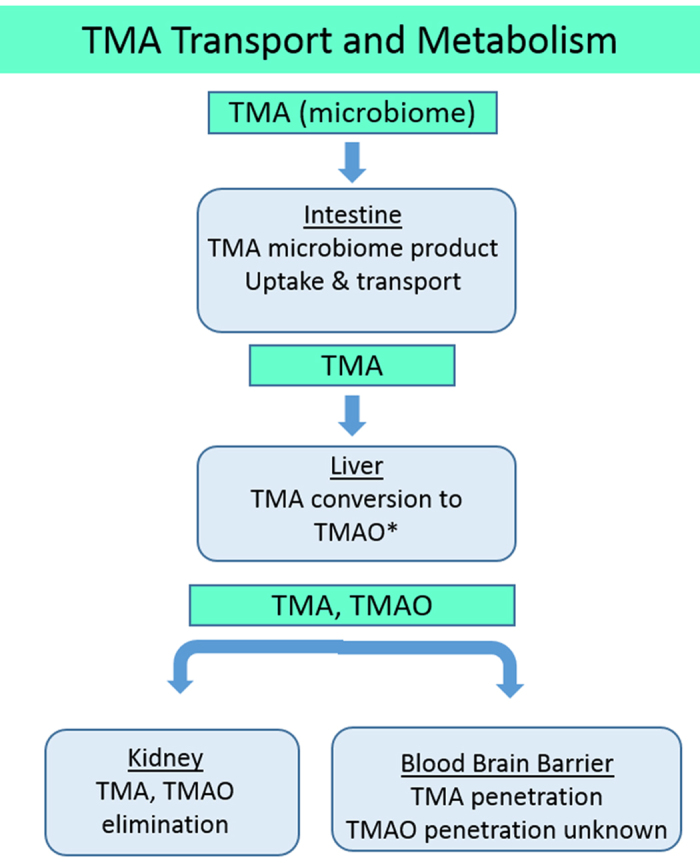
Functional analysis of TMA transport and metabolism in human organs. TMA produced in the intestine by the endogenous microbiome is taken up and transported to the liver, where it is metabolized to TMAO. Kidney disease can lead to increased plasma concentrations of TMAO, with significant medical side effects.

Figure 5 summarizes the *in vivo* uptake and metabolism of TMA and the transport and clearance of the TMAO metabolite. TMA hydrochloride at 316 μg/mL (3 mM) was added to the apical compartment of jejunal enteroids. After 24 h, 6% of the starting TMA concentration (19.1 ± 2.2 μg/mL) was found in the basolateral media (Supplemental Table S2). The basolateral media was subsequently diluted 1:3 in hepatocyte maintenance media and perfused into the SQL-SAL liver. Albumin synthesis confirmed liver function in the mixture of intestinal:hepatocyte maintenance media was not impacted (Supplemental Fig. S1A). The TEER results and restricted diffusion of 10 kD FITC-dextran polymer in the BBB were not impacted by the mixture of hepatocyte/EGM-2 media (Supplemental Information Supplemental Fig. S1 B,C). In the 24-h efflux media the TMA was below the detection limit and the TMAO metabolite concentration was 6.9 ± 0.3 μg/mL, confirming active liver metabolism. The liver-conditioned media was next diluted 1:3 in EGM-2 media or 1:2 in EGM-2 and perfused into the plasma side of the BBB/NVU and the vascular side of the VPTK module, respectively. After 12 h of perfusion through an intact BBB/NVU (Supplemental Fig. S1B,C), 4.6 ± 0.2 μg/mL TMAO was found in the vascular efflux and 1.8 ± 0.1 μg/mL TMAO in the neuronal efflux, equating to 26% TMAO penetration through the BBB (Supplemental Table S3), a novel finding. After 6 h of perfusion of liver-conditioned media through the VPTK model, the majority of TMAO (95%) was found in the vascular media while 5% was found in the lumenal media (Supplemental Table S2). An independent experiment was initiated in the vascularized kidney to measure levels of TMAO secreted using a media with higher concentration (375 μg/mL) TMAO. In this experiment 46% of the TMAO was found secreted into the kidney lumen.

### Four-organ coupling for vitamin D3 metabolites

**Figure 6:**
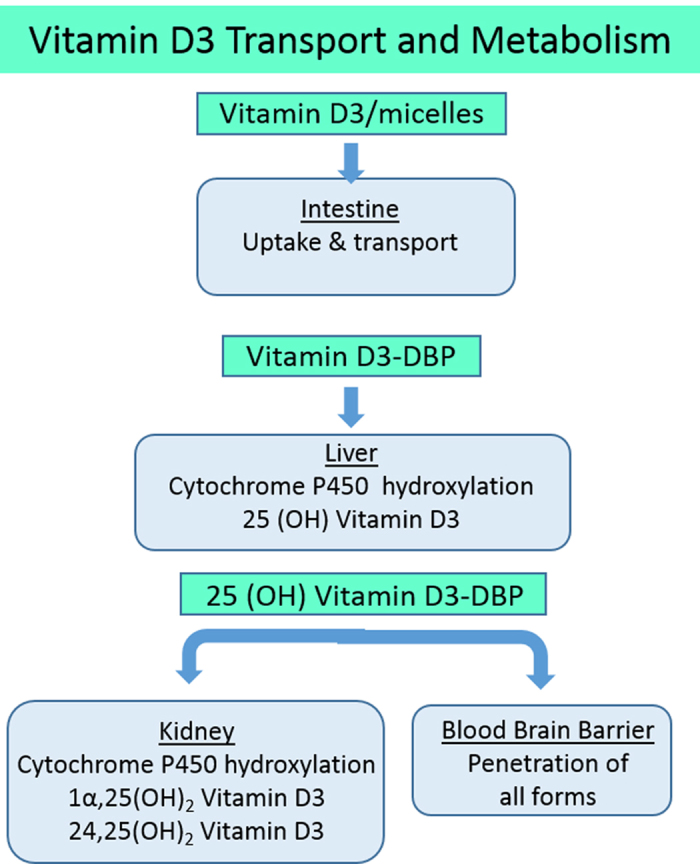
Functional analysis of Vitamin D3 transport and metabolism in human organs. Absorption and activation of Vitamin D3 requires transport and metabolism in multiple organs. Sequential metabolism in the liver and the kidney leads to a series of active metabolites. Vitamin D3 and its metabolites are hydrophobic and therefore can easily be lost in MPS systems due to binding to materials like PDMS. Use of a carrier protein is important in MPS devices for physiological relevance.

Figure 6 summarizes the *in vivo* uptake and metabolism of vitamin D3 and the transport and clearance of the vitamin D3 metabolites (see details in Supplemental Table S4). In these experiments, synthetic micelles^26^ containing 100 μM vitamin D3 were applied to the apical compartment of the differentiated human jejunal enteroids. Under these conditions 23% of the initial vitamin D3 concentration was transported to the basolateral media by 24 h (Supplemental Table S3). The basolateral media was then diluted 1:3 in hepatocyte maintenance media for treatment in the liver model. The liver model metabolized vitamin D3 to the 25-(OH) vitamin D3 metabolite, consistent with *in vivo* findings, although the kinetic rate was only 2.7% of *in vivo* rates. The concentration of 25-(OH) vitamin D3 in the liver-conditioned media collected during a 48-h or 120-h period, when diluted either 1:2 in kidney media or even at 4:1 in kidney media, was below the MS level of quantitation (Supplemental Table S3). However, approximately 6% of the 25-(OH) vitamin D3 infused into the vascular side of the BBB/NVU was found in the neuronal efflux media after 24 h. This would be consistent with the penetration of the 25-(OH) vitamin D3 through the BBB/NVU^27^.

Vitamin D3 is a fat soluble vitamin with a cLogP of 7.98, and in the body it is transported from the intestine in chylomicrons and systemically by vitamin D binding protein (DBP)^28^. DBP also binds to and transports the metabolites of vitamin D3. In the initial four-organ functional study, 80% of vitamin D3 perfused into the liver and 90% of the 25-(OH) vitamin D3 perfused into the kidney were unrecoverable. To determine if vitamin D3 loss was due to its high hydrophobicity, DBP was added to the perfusion media in the kidney and liver models. In the liver, inclusion of DBP resulted in over 50% recovery at 24 h and essentially 100% recovery from 24 to 48 h (Supplemental Table S5). In the kidney model, the use of DBP at the stoichiometric ratio of 3:1 vitamin D binding protein: 25-(OH) vitamin D3 improved recovery from 8% to 100% for a 48- to 72-h collection period (Supplemental Table S6). However, using the DBP did not lead to identification of the 1α, 25-(OH)_2_ vitamin D3 metabolite in the kidney efflux. In the liver experiments, the use of DBP at a stoichiometric ratio of 3:1 DBP: vitamin D3 improved recovery of vitamin D3 from 20% to 57% at 24 h, and to 100% at 48 h. In addition, the formation and recovery of the 25-(OH) vitamin D3 metabolite from the system was improved 2–3 fold (Supplemental Table S4).

### Summary of results

In summary, in the four-module functional coupling we found: (1) Terfenadine was metabolized to fexofenadine by the intestinal epithelium and liver; (2) Fexofenadine was a substrate for the intestinal epithelial counter-transport; (3) Fexofenadine cleared through the liver; (4) Fexofenadine was secreted into the kidney lumen; and (5) Fexofenadine did not penetrate the BBB. In concordance with known *in vivo* processing, TMA was transported across the jejunum, metabolized by the liver to TMAO and then secreted into the kidney lumen. Finally, vitamin D3 added to intestinal apical media in micelles was transported without metabolism across the jejunal monolayer, metabolized to 25-(OH) vitamin D3 by the liver model and penetrated the BBB model, parameters all consistent with oral uptake of vitamin D3. In the functionally coupled static liver and skeletal muscle myobundle models terfenadine toxicity was reduced by metabolism in the liver, consistent with clinical toxicity. These results were consistent with the known *in vivo* metabolism and transport of these compounds (Table 2), with a new finding that TMAO crosses the BBB.

## Discussion

An ultimate goal of this project and many other MPS organs-on-a-chip efforts is to develop a human experimental platform that could refine, reduce and eventually replace animal testing, by providing more relevant data that track human response and toxicity for development of therapeutics and prophylactics and the assessment of safety for industrial and environmental chemicals. Presently, individual human organ models are being developed in a variety of formats with increasing ability to recapitulate human organ functions. While it is important to optimize the performance of each individual organ, understanding the complexity of compound ADME/PK and toxicity in humans will eventually require coupling organ models. In this study we demonstrate that individual organ models can be functionally coupled to recapitulate sequential organ transport and metabolism as a precursor to physical coupling, for the refinement of the relative functional scaling of organ models, assessment of the compatibility of the materials and media, and demonstration that the coupled organ models recapitulate human *in vivo* ADME-TOX. We used the transport and metabolism of terfenadine, TMA and vitamin D3 in functionally coupled MPS models to demonstrate concordance with known *in vivo* processing. These studies demonstrate the organs will process test agents correctly during a single linear pass of media containing test agents in the presence of upstream organ metabolic products and secretions. The results provide a bench mark for future experiments which will directly couple the organs in a re-circulation media stream to mimic blood circulation in the whole body.

Human exposure to TMA occurs in industrial facilities where trimethylamine is manufactured or used, from tobacco smoke, and through the diet, but prominently is the result of the activity of the human intestinal microbiome, which can efficiently convert dietary choline and carnitine to TMA^29^. Increased blood concentration of TMAO is a predictive marker for risk of chronic renal disease, atherosclerosis and heart failure^30^,^31^,^32^.

Following uptake through the intestine, TMA is metabolized by the liver flavin-containing monooxygenase isoform 3 (FMO3) enzyme to the TMAO^33^. The four-organ model successfully demonstrated uptake of TMA, metabolism to TMAO and uptake by the kidney, suggesting the use of human-based MPS organ systems that include the microbiome, intestine, liver and kidney, for drug research targeting the microbiome production of TMA.

Terfenadine is a well-documented compound that was devised as an antihistamine (Seldane) but had serious off-target QT interval prolongation and fatal Torsades de Pointes through the inhibition of the delayed K rectified (hERG) channel in cardiac myocytes^34^,^35^,^36^. Terfenadine was removed from the market^37^,^38^,^39^ after researchers identified the cause of the toxicity as a drug-drug or diet-drug interference of CYP3A4 metabolism of terfenadine. They subsequently found that fexofenadine, the primary metabolite and active pharmacophore, had no effect on the hERG channel. In this report we confirmed that the functionally coupled organ models recapitulate several important qualitative PK parameters of terfenadine processing *in vivo*, suggesting that an optimally coupled MPS system, combined with computational modeling, could be used for predicting human PK and PD.

Terfenadine is known to bind to eag2 and inhibit the K + current in brain and skeletal muscle^22^. Terfenadine was shown to inhibit skeletal muscle myobundle tetanus and twitch response, except in the functionally coupled two-organ system when the liver was able to metabolize the parent compound to the non-toxic fexofenadine. These results demonstrate the potential for the use of coupled organs as toxicity models.

Vitamin D3 in humans comes from the conversion of 7-dehydrocholesterol in response to UVB radiation on the skin and through the diet^40^. Our initial experiments with vitamin D3 demonstrated that the jejunal enteroids did not metabolize the vitamin D3 during uptake via intestinal luminal micelles, which is the physiologically relevant result. The single hydroxylation step was observed to occur in the liver also as expected, but at a reduced rate compared to *in vivo* results. One plausible reason may be that the liver hydroxylation follows saturable, zero order kinetics at low concentrations. Vitamin D3 is also known to accumulate in fat when concentrations exceed the saturation limit^41^,^42^. Finally, the majority of the vitamin D3 and metabolites may have been “lost to the system,” presumably by binding to some of the device materials (e.g., PDMS), although some contribution from cell sequestration cannot be ruled out. Based on the vitamin D3 treatment passing organ to organ, however, we did confirm liver metabolism, BBB penetration and oral uptake.

After realizing the extensive vitamin D3 loss to some of the MPS materials, a second series of experiments was carried out including vitamin D binding protein (DBP) as a carrier in the liver and kidney models. DBP is an albumin-like protein that transports 85–90% of plasma 25-(OH) vitamin D3 in humans, with most of the remainder transported while bound to albumin^43^. The inclusion of DPB allowed nearly 100% recovery of 25-(OH) vitamin D3 from the syringe, tubing and device used in the kidney module. An additional recovery of 25-(OH) vitamin D3 was evident in the liver efflux, but whether this is simply due to increased recovery from binding or an actual improvement in liver metabolism cannot be determined from the study. We conclude that carrier proteins are essential components of models that include hydrophobic biomolecules like vitamin D3, and may be a viable approach to testing more hydrophobic compounds in MPS systems where lower drug binding materials are not yet available. The advantage of using carrier binding proteins may be limited to compounds with known binding characteristics, such as pharmaceutical compounds; however, it may also apply to many environmental toxins and the majority of the untested 80,000 commonly used chemicals, which will be addressed in a follow-up study. It is important to note that the use of carrier proteins in MPS experiments may be required for proper recapitulation of human physiology and toxicology, since these proteins are critical for the delivery of endogenous high logP hormones, metabolites, and toxins in the normal and diseased human.

In addition to the concordance of the findings in this study to known *in vivo* processing, the study also served to identify some challenges to directly coupling organ systems in order to realize a multi-organ “human on a chip”. Although many of these biological and technical challenges, listed in Tables 3 and 4, were anticipated^19^, the experiments allowed us to refine our understanding of their importance and difficulty.

Organ-to-organ scaling is a complex issue that is not readily solved simply by allometric scaling^20^. For example, the liver in these studies is approximately 1/3 of a micro-human by allometric scaling and the proximal tubule kidney is only 1/10 of a micro-human. However, the metabolic conversion of vitamin D3 by the liver did not produce enough vitamin D3 metabolite to demonstrate the kidney hydroxylation step within the limits of the mass spectrometry quantitation^20^ (Table 2, Supplemental S4). This could mean that the production of metabolite in the liver was not sufficient, the metabolism in the kidney was not sufficient, there was too much media dilution, or the metabolites were not transported effectively from the liver to the kidney. This experiment highlights the importance of functional scaling, which can be established in isolated systems, rather than relying on allometric scaling to select the proper relative sizes of organ models.

A major challenge in coupling organs is addressing the custom media formulations developed and optimized for each organ. For functional coupling we chose to mix effluent from one organ with the optimal media for the next organ to lessen the impact of metabolic waste products, and to ensure that essential components of the optimal media were included for each organ model. One of the interesting results from this study is that the organ systems seemed to tolerate mixtures of different media and media supplements for at least 24 to 72 h. That suggests that a universal media may be a reasonable goal. It should also be noted that vascularization of organ models will facilitate the development of a universal media by providing an endothelial barrier between the circulating perfusate and the stromal cells of each organ, and therefore is seen as a priority in future model development.

Several technical hurdles, listed in Table 4, need to be addressed before physically coupled organ models will be widely adopted for use in ADME/PK and analytics modeling.

Although the drug and hormone binding properties of PDMS have been known and reported for years^44^,^45^,^46^, they are often ignored in lieu of the ease of device production. These studies, with a modestly hydrophobic drug and a common fat soluble vitamin, highlight the potential depth and severity of drug loss to the standard materials, disposable syringes and tubing often used in static and microfluidic systems (Supplemental Fig. S2). Drug loss can be minimal with some compounds (e.g., compounds with low cLogP) and near 100% for others (supplemental results Fig. 3). Current research into novel solutions includes the development of coatings, alternative plastics with low drug binding properties^47^,^48^,^49^,^50^ and the potential use of vascularization to isolate drug delivery from the device materials. Even if some or all of these solutions are realized, an additional physiological approach to processing hydrophobic compounds is the use of carrier proteins. In the four-organ vitamin D3 experiments, we found that including a stoichiometric amount of DBP allowed the hydrophobic vitamin D3 to be transported and metabolized in the SQL-SAL and PTK models. We plan to test this strategy with other hydrophobic compounds and anticipate adding a general carrier protein to our media formulation in future models.

Although not specifically addressed in this study, oxygenation of coupled organ models is expected to be a significant technical challenge. The functional coupling approach used in this study allowed for media equilibration with atmospheric oxygen between organs. Physically coupled organ models will have to address oxygenation, presumably through permeability of materials and/or the use of local recirculation reservoirs that can be selectively oxygenated or deoxygenated. Although PDMS is a problem when it comes to binding hydrophobic compounds, the high rate of diffusion of oxygen in PDMS has been an aid to oxygenation. However, it is important to model and measure oxygen tension in devices to be sure that oxygen tension is appropriate for the tissue, and not depleted by consumption by the cells^51^. This can be a significant challenge in some organ models such as the intestine, where one side should be anaerobic like the intestinal lumen, while the other side should be oxygenated. Materials that allow maintenance of physiologically relevant, experimentally established gas gradients over the time of the experiment are an area that requires development.

In these studies, platforms were developed independently for use as individual organ models and integration was by achieved by transport of organ efflux. These studies demonstrate the potential for the use of functionally coupled multi-organ human MPS for ADME/TOX and PK assessment, and to provide reference data for the validation of directly coupling of organ systems. Optimally coupled organ models that can demonstrate ease of use, reasonable cost and reliability to stakeholders in industry and academia will be widely adopted to answer questions on human PK, dose-limiting organ toxicity, pharmacodynamics (PD) and mechanisms of disease progression.

## Methods

A summary of methods used for the preparation and functional coupling in the four-organ and two-organ experiments follows. More detailed methods and dimensions of the individual devices are provided in the Supplemental Material and Supplemental Table S7. The human liver, kidney, NVU and skeletal muscle organ constructs have established long term viability for at least 28 days in culture^52^,^53^,^54^. The human intestine enteroid transwells were used within 24 h but have been reported to maintain positive barrier functions at least to 72 h^55^.

### Mass Spectrometry Analysis

Mass spectrometry was used to measure the concentration of compound and metabolites in the media following exposure in each MPS organ module. Details of the instruments and protocols are summarized in Supplemental Table S1. All Vitamin D3 samples were analyzed at U Washington using an established method^52^,^56^. All TMA, TMAO samples were analyzed at U Washington using a novel LC MS/MS method (unpublished). Terfenadine/fexofenadine mass spectrometry analyses were conducted at UPitt, U Washington, Duke and Vanderbilt using previously published methods^57^,^58^.

### Preparation of Intestinal MPS Module

Human jejunal enteroid cultures were isolated from two separate donors as described^59^ and maintained in culture as spherical enteroids in Matrigel (Becton Dickinson, Franklin Lakes, NJ) until dissociated and plated as monolayer cultures on collagen IV coated polyethylene terephthalate (PET) Transwell^®^ membrane inserts (Corning, Inc., Corning, NY) (Fig. 1A)^55^,^59^,^60^,^61^. The apical chamber received 100 μl of a 5 × 10^6^/mL of the suspended cells in proliferation media. The basolateral chamber received 600 μl of proliferation media. After 24 h (terfenadine, TMAO experiments) the cells were switched to differentiation media and incubated for an additional 4–5 days post-differentiation, with media changes every 2 days. In other experiments (Vitamin D3 uptake experiments), the monolayers were monitored until confluence achieve based on transepithelial electrical resistance (TEER) and then the cells were switched to differentiation media for an additional 5 days of differentiation. Jejunal barrier monolayer with intact tight junctions were established by TEER testing and the restricted diffusion of 4 kD FITC-dextran polymers over the 24 h period (Supplemental Information Supplemental Table S8).

### Preparation of the Liver MPS Modules

The sequentially layered self-assembly liver module (SQL-SAL) used for terfenadine and TMA experiments was constructed in a Nortis (Seattle, WA) microfluidic device as previously described (Fig. 1B)^53^. Media without dexamethasone was used to avoid downstream toxicity to the human neurons in the NVU. For the initial vitamin D3 experiments, the 4-cell static SQL-SAL model was constructed in 96-well microplates to avoid the absorption of vitamin D3 and its metabolites by the PDMS in the Nortis devices (see Supplement for additional details). Media samples were collected after 24 h and 48 h incubations.

### Preparation of Vascularized Proximal Tubule Kidney (VPTK) MPS Module for Terfenadine and TMA Exposure

To form the vascularized kidney model, 2 lumens were pre-cast side-by-side in gelling collagen inside the Nortis microfluidic device. Human proximal tubule epithelial cells (PTEC) were seeded on the wall of one lumen channel at an approximate density of 20 × 10^6^ cells/mL and were allowed to attach for 1 h. Culture media flow at 30 μl/h was then initiated and maintained for 5 days in order to establish a 100% confluent tubule expressing tight junctions. On day 6, the flow of culture media to the PTEC channel was stopped to allow the second channel seeding at 20 × 10^6^ human umbilical vein endothelial cells/mL (HUVEC) and allowed to attach for 30–60 minutes. Parallel media flow of defined DMEM/F-12 (see Supplement) with 2% FBS for the PTEC channel and EGM-2 media for HUVEC channel were initiated at a rate of 30 μl/h. The VPTK devices were allowed to stabilize overnight before conducting test compound treatment.

### Preparation of the Proximal Tubule Kidney (PTK) Module for Vitamin D3 Exposure

Commercial Nortis microfluidic devices seeded with PTEC were used in vitamin D3 experiments (Fig. 1C). Briefly, a single 100 μm diameter lumen was pre-cast in gelling collagen inside the microfluidic device and then seeded with passage 5 kidney PTEC to 100% confluence according to established methods^52^. The kidney proximal tubule modules were then perfused (30 μL/h) for 8 days with 100% kidney proximal tubule media prior to perfusion with mixed media solutions.

### Test of Vitamin D Binding Protein as a Carrier in the PTK Module

In order to further refine the PTK module for the quantitation of the metabolism of vitamin D and other highly adsorptive compounds, FBS and purified human vitamin D binding protein (DBP) were evaluated as vitamin D delivery vehicles. Eight kidney PTK modules were seeded to 60–90% confluence with human PTECs (passage 3) as above. The module was perfused with kidney proximal tubule media at a rate of 30 μL/h. After 6 days in culture, the PTK modules were randomly divided into 2 groups (n = 4 for each group). The media for one group was then supplemented with 1 μM 25-(OH) vitamin D3 and either 2% v/v FBS or 3 μM human DBP. Media efflux was collected for the time interval of 48 to 72 h following the initiation of treatment. Media was also collected on day 3 from the input for assessment of 25-(OH) vitamin D3 adsorption to the syringes and input tubing. All media samples were stored in amber glass vials at −80 °C until LC-MS/MS analysis.

### Preparation of the Blood-Brain Barrier/Neurovascular Unit

The BBB/NVU device was constructed as previously described^54^ (Fig. 1D). Briefly, before introducing cells, the device was coated with 9.6 μg/mL laminin for 24 h at 37 °C. On Day 0, primary human brain-derived microvascular endothelial cells (hBMVEC) were loaded into the vascular chamber (1 × 10^6^ cells/mL), followed by device inversion to allow cell attachment to the membrane. On Day 1, media perfusion at 2 μL/min was initiated, and the hBMVECs were allowed to grow for 12 days to reach confluence and establish tight junctions. On Day 12, the device was turned over and a 2:1 mix of primary human astrocytes and pericytes was loaded into the brain chamber and allowed 1–2 days to reach confluence. On Day 14, collagen gel (1.75 mg/mL) containing human iPSC-derived neurons and co-differentiating astrocytes (4 × 10^6^ cells/mL) was loaded on top of the astrocytes and pericytes. The collagen gel was given 2 h to solidify before restarting perfusion of the brain chamber. During the first 3 days after the neurons were added, the brain chamber was perfused with media containing Rho-associated coiled-coil kinase (ROCK) inhibitor (10 μM, Tocris) Y-27632 (Sigma-Aldrich, St. Louis, MO, USA) to help the neurons survive the stress of re-plating^62^,^63^,^64^,^65^.

### Preparation of the Static Hepatocyte and Skeletal Muscle Myobundles Modules

Human skeletal muscle myobundles were prepared as described by Madden *et al*.^25^ and cultured in human growth media containing 1.5 mg/mL aminocaproic acid on a rocker (0.33 Hz) at 37 °C. On day 4, the media was switched to differentiation medium consisting of low glucose DMEM supplemented with 2% adult horse serum (HS), 100 μM fatty acids (1:1 oleate:palmitate) conjugated to 0.14% BSA, 100 μM carnitine, 2 mg/mL of ACA, and 1x antibiotic-antimycotic. Myobundles were incubated for 2 weeks prior to compound exposure on a rocker at 0.33 Hz.

Fresh primary human hepatocytes (Triangle Research Labs, TRL; Durham, NC) were cultured in a collagen sandwich culture^66^ under static conditions at 6.5 × 10^5^ cells/mL in 24-well collagen coated plates. One day after preparation in plating media (TRL MP250) the cultures were switched to maintenance media (TRL MM250) and incubated for three days prior to compound exposure for 24 h.

### Test Compound Preparation

Terfenadine at a final 10 μM concentration was prepared by diluting 1:1000 a stock 10 mM/DMSO into intestinal apical media. TMA was prepared at a concentration 3 mM by dissolving the neat compound directly into intestinal apical media. Vitamin D3 containing micelles was prepared by mixing 0.04 mM phosphatidylcholine, 0.3 mM monoolein, 5 mM sodium taurocholate, and 100 μM vitamin D3 in a glass tube and evaporating the solvents under N_2_ gas^26^. The dried residue was solubilized in apical media and mixed by sonication in a water bath for 30 s.

### Four-Organ Functional Coupling Experiments

Figure 2 illustrates the work flow between the respective laboratories for functional coupling of 4 organ modules. All treatments were conducted in duplicate modules in each laboratory. Jejunum intestinal Transwell modules were set up at Baylor and JHU for exposure to 10 μM terfenadine, 100 μM vitamin D3 or 3 mM TMA in the apical media. TER levels in the intestine modules were found to be acceptable for all test agents. The apical and basolateral media were collected in glass vials and frozen at −80 °C for MS analysis and transported to UPitt for testing in the SQL-SAL module.

For testing in the SQL-SAL modules, the intestinal media samples were thawed, diluted 1:3 in naïve liver media and then either perfused through the SQL-SAL module for 24 hr (terfenadine and TMA-conditioned media) or incubated for 72 h in the static SQL-SAL in 96-well microplates (Vitamin D3-conditioned media). The intestinal/liver-conditioned media efflux was collected and samples were tested by MS or transported on dry ice for testing in the kidney module, or the BBB/NVU module.

For testing terfenadine and TMA/TMAO in the VPTK module, a 1:4 mixture of intestine/liver-conditioned media and naïve EGM-2 media was perfused through the HUVEC channel. The media for the PTEC channel remained unchanged. Media perfusion was initiated simultaneously for both channels at a flow rate of 60 μl/h. The effluents from both channels were collected for 6 h. Due to the insensitivity of the TMAO analyte by mass spectrometry, an additional TMAO experiment using EGM-2 media supplemented with 5 mM TMAO was conducted in the VPTK to quantitate the level of TMAO secretion.

For testing Vitamin D3 in the PTK module, either a moderate 1:2 or high 4:1 dilution of liver-conditioned:naïve kidney media was used. The mixed media solutions were perfused into individual proximal tubule kidney MPS modules at 15 μL/h. The media efflux was collected from 0 to 48 h and from 48 to 120 h to assess steady-state disposition of vitamin D metabolites. All media samples were stored in amber glass vials at −80 °C until LC-MS/MS analysis. Samples were analyzed using an established method for assessing vitamin D3, 25-(OH) vitamin D3, 24,25-(OH)_2_ vitamin D3, 4β,25-(OH)_2_ vitamin D3 and 1α,25-(OH)_2_ vitamin D3^52^ (Supplemental Table S4).

For treatment in the BBB/NVU module, the combined intestinal/liver-conditioned media was diluted 1:3 in blank EGM-2 vascular compartment media.

### Functional Coupling of the Static liver with Skeletal Muscle for Terfenadine Toxicity Testing

For compound treatment, the static liver model was dosed daily with fresh 10 μM terfenadine. Liver function was assessed by measuring CYP3A4 activity using luminescence (Promega) and normalized lactate dehydrogenase (LDH) levels (ThermoFisher). For treatment, myobundles were switched to the media conditioned in the static liver model with or without terfenadine. Normalized LDH levels were measured in the skeletal muscle media to assess cell death.

Myobundles were loaded into a custom-made force measurement apparatus containing a sensitive optical force transducer and a computer-controlled linear actuator (ThorLabs), as previously described^25^. Samples were stimulated (40 V/cm) to recreate twitch (1 Hz for 10 ms) and tetanic (20 Hz for 1 s) contraction. Contractile force traces were analyzed for peak twitch and tetanus force using a custom MATLAB (Mathworks) program.

### Study Approvals

All experiments using human samples and human tissues were performed in accordance to relevant guidelines and regulations. Experiments were approved by the following University regulatory bodies: Duke University Institutional Review Board; University of Pittsburgh Institutional Biosafety Committee and human Stem Cell Research Oversite Committee; Baylor College of Medicine Institutional Review Board; University of Washington Institutional Human Subjects Board; Johns Hopkins University Medicine Institutional Review Board; Vanderbilt University Institutional Review Board. Skeletal muscle tissue was obtained from surgical waste and is considered exempt by the Duke University Institutional Review Board. No informed consent was required. Informed consent was obtained from all other subject procurement of tissue obtained in this report.

## Additional Information

**How to cite this article:** Vernetti, L. *et al*. Functional Coupling of Human Microphysiology Systems: Intestine, Liver, Kidney Proximal Tubule, Blood-Brain Barrier and Skeletal Muscle. *Sci. Rep.* **7**, 42296; doi: 10.1038/srep42296 (2017).

**Publisher's note:** Springer Nature remains neutral with regard to jurisdictional claims in published maps and institutional affiliations.

## Supplementary Material

Supplemental Material

## Figures and Tables

**Table 1:** Microphysiological Systems Used for Functional Coupling.

	Intestine	SQL-SAL Liver	BBB/NVU	Proximal Tubule Kidney
Cell Types	Differentiated human jejunal enteroids^a^	Primary hepatocytes, Ea.Hy926 endothelial, macrophage immune^b^, LX-2 stellate	Neuronal: iPSC-derived human neurons, pericytes, astrocytes Vascular: HBMVEC^c^	PTK: PTEC^d^ VPTK: HUVEC^e^, PTEC
Model Type Media volume /24 h	Transwell (Static) 100/600 μl apical/basolateral	MPS MPS: 120 μl Static: 50 μl/well	MPS Vascular: 1440 μl Neuronal: 1440 μl	MPS PTK: 720 μl VPTK: 720 μL
Sampling: Terfenadine TMA Vitamin D3	24 h 24 h 24 h	24 h 24 h 72 h	24 h 12 h 24 h	VPTK: 6 h VPTK: 6 h (Vas. PTC) PTK: 0–48; 48–120 h
Media	EM	HMM	Vascular Media Neuronal Media	PTEC Vas. PTEC
Functional Coupling Media	**Compound Perfusion Media** EM -Wnt3a	**Perfusion Media** 33% conditioned 67% HMM minus dexamethasone	**Vascular Compartment**33% Conditioned 67% EBM-2, 5% FBS **Neuronal Compartment** 99% EBM-2, 1% FBS, B27^®^	**Proximal Tubule** 50% Conditioned 50% DMEM/F12 2% FBS **Vascular** 25% Conditioned 75% EGM-2

EM: Advanced DMEM/F12 + Wnt3a, R-spondin 1, Noggin, EGF/EM minus Wnt3a.

HMM: Williams E, 1.25 μg/ml albumin, 100 ng/ml insulin, 100 nM dexamethasone.

^a^Mature enterocytes enteroendocrine, and goblet cells.

^b^PMA differentiated U937 cells.

^c^Human brain microvascular endothelial cells.

^d^Primary human kidney proximal tubule epithelial cells.

^e^Human umbilical vein endothelial cells.

**Table 2:** Key Concordances Between MPS and Clinical Fate for Three Test Agents.

Test Agent/*Metabolites*	Clinical MPS Model	Intestine	Liver	Kidney	BBB
**TMA** *TMAO*	**Clinical**	Uptake & Transport	**TMA** → *TMAO* < 5% **TMA** Clearance	> 95% *TMAO* Excreted	*TMAO* Penetration: Unknown
	**MPS**	Uptake & Transport	**TMA** → *TMAO* < 1% TMA Clearance	~46% *TMAO* Excreted	26% *TMAO* Penetration
**Terfenadine (Ter)** *Fexofenadine (Fex*)	**Clinical**	**Ter** → *Fex*; *Fex* CounterTrans	< 1% Bio T < 95% *Fex* Clearance	11% *Fex* Excreted	~0% *Fex* Penetration
	**MPS**	**Ter** → *Fex*; *Fex* CounterTrans	< 1.4% Bio T (est.) < 80% *Fex* Clearance	~ 1% *Fex* Excreted	~ 0% *Fex* Penetration
**Vitamin D3 (VD3)** *25(OH)VD3; 1α,25(OH)*_*2*_*VD3; 24,25(OH)*_*2*_*VD3*	**Clinical**	Uptake & Transport No metabolism	**VD3** → *25(OH)VD3*	*25(OH)VD3 *→* 1α,25(OH)*_*2*_*VD3 & 24,25(OH)*_*2*_*VD3*	**VD3** & 2*5(OH)VD3* Penetration
	**MPS**	Uptake & Transport No metabolism	**VD3** → *25(OH)VD3 & 24,25(OH)*_*2*_*VD3*	*1α,25(OH)*_*2*_*VD3 & 24,25(OH)*_*2*_*VD3* below LOQ	0.4% **VD3** & 6% *25(OH) VD3* Penetration

Key: Uptake - by jejunum endothelial cells; Transport - from apical to basolateral media; → = Metabolism; CounterTrans = Transport from basolateral to apical media; est. = estimated.

Excreted - into proximal tubule lumen; *LOQ* = limit of quantitation; Penetration - through blood-brain barrier.

**Table 3:** Key Biological Challenges For Direct Physical Coupling 4 MPS Organs.

Key Biological Challenges
Implementing a universal medium
Proper scaling of MPS models to reproduce maximal functions
Further developing intestine in MPS device
Implementing a combination of real-time fluorescent labeled biosensors in multiple organs
Establishing a source of renewable adult iPSC for all/most cell types to provide single donor disease phenotype cells and to overcome current need to mix primary cells or established cell lines from genetically diverse sources
Vascularization of all MPS models

**Table 4:** Key Technical Challenges For Direct Coupling 4 MPS Organs.

Key Technical and Platform Integration Challenges
Minimizing drug/biomolecule binding to PDMS, tubing, membranes and devices made from various materials
Minimizing connection volumes and bubbles while maintaining sterile conditions
Creating oxygenation conditions for each organ, including different oxygenation on apical and basolateral surface of intestine and zonation in the liver
Creating optimal flow rate in each MPS organ
Establishing PK analytics and modeling from data captured in database^67^
Integrating dynamic, chemical and electrical cues, including contributions of missing organ systems
