# Corrigendum: Functional Coupling of Human Microphysiology Systems: Intestine, Liver, Kidney Proximal Tubule, Blood-Brain Barrier and Skeletal Muscle

**DOI:** 10.1038/srep44517

**Published:** 2017-03-16

**Authors:** Lawrence Vernetti, Albert Gough, Nicholas Baetz, Sarah Blutt, James R. Broughman, Jacquelyn A. Brown, Jennifer Foulke-Abel, Nesrin Hasan, Julie In, Edward Kelly, Olga Kovbasnjuk, Jonathan Repper, Nina Senutovitch, Janet Stabb, Catherine Yeung, Nick C. Zachos, Mark Donowitz, Mary Estes, Jonathan Himmelfarb, George Truskey, John P. Wikswo, D. Lansing Taylor

Scientific Reports 7: Article number: 4229610.1038/srep42296; published online: 02 08 2017; updated: 03 16 2017

The original version of this Article contained an error in Affiliation 2, which was incorrectly given as:

‘Department of Computational and Systems Biology, University of Pittsburgh, Baltimore, PA, USA’.

The correct affiliation is listed below:

‘Department of Computational and Systems Biology, University of Pittsburgh, Pittsburgh, PA, USA’.

Additionally, there was a typographical error in the Acknowledgements section.

‘EPA STAR 8357360 (UPitt)’.

now reads:

‘EPA STAR 83573601 (UPitt)’.

These errors have been corrected in both the PDF and HTML versions of the Article.

