# Supplementary material for: Functional Coupling of Human Microphysiology Systems: Intestine, Liver, Kidney Proximal Tubule, Blood-Brain Barrier and Skeletal Muscle
Source: Sci Rep. 2017 Feb 8;7:42296. doi: 10.1038/srep42296 (PMC5296733; doi:10.1038/srep42296)
Supplement: Supplemental Material [file srep42296-s1.doc]

**Supplemental Material to:**

**Functional Coupling of Human Microphysiology Systems: Intestine, Liver, Kidney Proximal Tubule, Blood-Brain Barrier and Skeletal Muscle.**

Lawrence Vernetti*^#^,^1,2^ Albert Gough^#^,^1,2^ Nicholas Baetz,^3^ Sarah Blutt^4^, James R. Broughman^4^, Jacquelyn A. Brown,^5^Jennifer Foulke-Abel,^3^ Nesrin Hasan,^3^ Julie In,^3^ Edward Kelly,^6^ Olga Kovbasnjuk,^3^ Jonathan Repper,^7^ Nina Senutovitch,^1^ Janet Stabb,^3^ Catherine Yeung,^8,9^ Nick C. Zachos,^3^ Mark Donowitz,^3†^ Mary Estes,^5†^ Jonathan Himmelfarb,^9,10†^ George Truskey,^11^ John P. Wikswo,^4,12,†^ and D. Lansing Taylor^1,2,13†^

1-University of Pittsburgh Drug Discovery Institute, 2-Dept. of Computational and Systems Biology, University of Pittsburgh, 3- Departments of Physiology and Medicine, GI Division, Johns Hopkins University School of Medicine, 4- Departments of Molecular Virology and Microbiology and Medicine, Baylor College of Medicine, 5- Department of Physics and Astronomy, Vanderbilt Institute for Integrative Biosystems Research and Education, Vanderbilt University, 5-Baylor College of Medicine, 6- Department of Pharmaceutics, University of Washington, 7- Department of Biomedical Engineering, Duke University, 8 -Department of Pharmacy, University of Washington, 9- Kidney Research Institute, University of Washington 10- Department of Medicine, University of Washington, , 12- Department of Biomedical Engineering, Vanderbilt University, 13-University of Pittsburgh Cancer Institute.

#These authors contributed equally to this work; †These senior authors contributed equally to this work;*corresponding author

### Supplemental Table S1. Mass Spectroscopy Test Systems and Conditions

| **Laboratory** | **Compounds** | **Test System** | **Gradient (time in min)** |
| --- | --- | --- | --- |
|  |  | **Detector** |  |
| **University of Pittsburgh** | Terfenadine/  Fexofenadine | Waters Acquity UPLC BEH C18, 1.7 µm, 2.1 X 100 mm reverse - phase column | 0.0 - 0.5: Mobile phase start:  20% ACN/W/0.1% FA  0.5 - 2.5: gradient to 95% ACN  2.5 - 3.5: hold at 95% ACN  3.5 - 4.0: gradient to 20% ACN |
|  |  | ESI, Thermo Fisher TSQ Quantum Ultra Mass Spectrometer |  |
| **Vanderbilt University** | Terfenadine/  Fexofenadine | Waters Acquity UPLC HSS T3 C18, 1.8 um, 1.0 X 100 mm reversed-phase column | 0 - 1 : 1% ACN  1 - 10: gradient to 50% ACN  10 – 15: gradient to 70% ACN  15 – 20: gradient to 99% ACN  20 – 22: hold at 99% ACN  22 – 25: gradient to 1% ACN  25 - 30: equilibrate at 1% ACN |
|  |  | ESI, Waters, Synapt G2, Ion Mobility Mass Spectrometer |  |
| **Duke University** | Terfenadine | Waters Acquity UPLC BEH C18 1.7 µm, 2.1x50 mm reverse-phase column | 0.0 - 4.0: Mobile phase start:  20% ACN/W/0.1% FA  4.0 - 6.5: gradient to 36% ACN/0.1% FA  6.5 - 9.1: gradient to 100% ACN/0.1% FA  9.1 - 9.5: Hold  9.5 - 12.6: gradient to 20% ACN/0.1% FA |
|  |  | ESI, Thermo Fisher TSQ Quantum Ultra Mass Spectrometer |  |
| **University of Washington** | TMA/TMAO | WATERS Atlantis HILIC 3 um,  2.1 x 100 mm column | A: 100 mM AA  0.0 – 2.0: Mobile Phase Start:  5% AA/87.5% ACN/7.5% H  2.0 - 7.5: gradient to 5% AA/60% ACN/35% W  7.5 - 9.5: hold 5% AA/60% ACN/35% W  9.5 - 14: gradient to 5% AA/87.5% ACN/7.5% W |
|  |  | ESI, Thermo Fisher TSQ Quantum Ultra Mass Spectrometer |  |
| **University of Washington** | Vitamin D3, hydroxylated metabolites; | reference 53 | reference 53 |
| AA- NH_4_COOH/0.4% HCOOH pH 3.6, ACN- acetonitrile, FA- , H- , W- water | | | |

### Supplemental Table S2. Terfenadine and Fexofenadine Results in 4 Functionally Coupled MPS Systems

| **Organ** | **Collection interval** | **Terfenadine (ng/ml)** | **Fexofenadine (ng/ml)** |
| --- | --- | --- | --- |
| **Intestine**  Apical Media  Basolateral Media | 0-24 h | 389.4 ± 62.2  53.7 ± 28.0 | 44.4 ± 7.3  26.4 ± 3.5 |
| **Liver**  Efflux Media | 0-24 h | 0.1 ± 0.1 | 21.4 ± 3.4 |
| **Brain**  Vascular Efflux  Neuronal Efflux  Vascular Efflux Spiked Sample^3^ Neuronal Efflux | 0-24 h | 0.1 ± 0.7  < LOD^1^  25.8 ± 1.3 11.7 ± 1.6 | 4.1 ± 0.3  < LOD  8.9 ± 0.2  0.4 ± 0.2 |
| **Kidney**  Plasma Efflux  Proximal Tubule Lumen | 0-6 h | < LOQ^2^ < LOQ | 10.2 ± 3.6 0.1 ± 0.1 |

1. LOD- Level of Detection

2. LOQ- Level of Quantitation

3. EGM-2 media with 30 ng/ml terfenadine and 50 ng/ml fexofenadine

# Supplemental Table S3. TMA, TMAO Results in 4 Functionally Coupled MPS Systems

| **Organ** | **Collection interval** | **TMA (ng/ml)** | **TMAO (ng/ml)** |
| --- | --- | --- | --- |
| **Intestine** Apical Media  Basolateral Media | 0-24 h | 3.41 X 10^5^  19100 ± 2179 | < LOQ^1^  < LOQ |
| **Liver** Efflux Media | 0-24 h | < LOQ | 6900 ± 300 |
| **Brain (12 h)** Vascular Efflux Neuronal Efflux | 0-24 h | < LOQ  < LOQ | 4600 ± 200 1800 ± 100 |
| **Kidney Perfused with Liver Conditioned Media**  Plasma Efflux  Proximal Tubule Lumen | 0-6 h | < LOQ  <LOQ | 21720 ± 6967  450 ± 290 |
| **Kidney Perfused at 3.75 X 10^5^ ng/ml TMAO Spiked Media**  Plasma Efflux  Proximal Tubule Lumen | 0-6 h | < LOQ < LOQ | (2.16 ± 1.19) X 10^5^  (1.84 ± 1.25) X 10^5^ |

### Supplemental Table S4. Vitamin D3 and Metabolite Results in 4 Functionally Coupled MPS Systems

| **Organ** | **Vitamin D3**  **(ng/ml)** | **25-(OH) Vitamin D3 (ng/ml)** | **1α,25-(OH)_2_ Vitamin D3 (ng/ml)** | **4β,25-(OH)_2_ Vitamin D3 (ng/ml)** | **24,25-(OH)_2_ Vitamin D3 (ng/ml)** |
| --- | --- | --- | --- | --- | --- |
| **Intestine**  Micelle  Apical Media Basolateral Media | 36000 ± 4200 20000 ± 2200 8300 ± 410 | < LOQ^1^ < LOQ  < LOQ | < LOD^2^ < LOD  < LOD | < LOD^2^  < LOD  < LOD | < LOD^2^ < LOD  < LOD |
| **Liver** Efflux Media | 1100 (pooled)^3^ | 3.0 | 0.0064 | 0.047 | 0.25 |
| **Brain** Vascular Efflux Neuronal Efflux | 45 ± 6.2  0.19 ± 0.23 | 0.82 ± 0.15  0.051 ±0.022 | < LOD  < LOD | 0.0081 ±0.0029  < LOD | 0.079 ±0.015  < LOQ |
| **Kidney**  Efflux Media (0-48 h):  50% Liver-conditioned  80% Liver-conditioned  Efflux Media (0-120 h):  50% Liver-conditioned  80% Liver-conditioned | 1.5^4^  4.3  1.0  1.4 | 0.12  0.23  0.29  0.48 | < LOD  < LOD  < LOD  < LOQ | 0.0033  0.0064  0.0076  0.014 | 0.082  0.12  0.11  0.16 |

1. LOQ Limit of Quantitation
2. LOD Limit of Detection
3. Vitamin D3 conditioned in 4-cell liver module constructed in a 96 well plate. All media pooled to provide the needed volume for the kidney and neurovascular modules.
4. N=2

### Supplemental Table S5. Effect of Vitamin D Binding Protein on Recovery of Vitamin D3 and Metabolites from the Liver MPS Organ Module

| **Liver MPS**  **Perfusion Media**  **7 μM VitD3** | **Collection Interval (h)** | **Hepatocyte Williams E Media + 1.25 mg/ml BSA** | **Hepatocyte Williams E Media, 1.25 mg/ml BSA, 3 μM Vitamin D Binding Protein** |
| --- | --- | --- | --- |
| **VD3 Recovered in Efflux Media** | 0-24 | 20% recovery | 57% recovery |
|  | 24-48 | Not measured | 100% recovery |
| **25-(OH) VD3**  **(pmol/day/10^6^ Hepatocytes)** | 0-24 | 7.00 (2.7% of *in vivo* rate) | 12.63 (4.9% of *in vivo* rate) |
|  | 24-48 | Not measured | 24.38 (7.8% of *in vivo* rate) |

### Supplemental Table S6. Effect of Vitamin D Binding Protein on Recovery of Vitamin D3 and Metabolites from the Kidney MPS Organ Module

| **Kidney MPS Perfusion Media**  **1 μM 25-(OH) VD3** | **Collection Interval**  **(h)** | **Renal Tubular Epithelial Media + 2% FBS** | **Renal Tubular Epithelial Media + 3 μM Vitamin D Binding Protein** |
| --- | --- | --- | --- |
| **25-(OH) VD3** | 48-72 | 8% recovery^1^ | 100% recovery |
| **1α,25-(OH)_2_ VD3 (pmol/day/ 10^6^ Proximal Tubule Cells)** | 48- 72 | < LOQ^2^ | < LOQ |
| **24,25-(OH)_2_ VD3 (pmol/day/ 10^6^ Proximal Tubule Cells)** | 48- 72 | 6.80 ± 1.6 | - 1. ± 2.2 |

1. 16% recovered from syringes and tubing leading up to MPS module
2. LOQ - Limit of Quantitation

**Supplemental Table S7. Source, Area Shape, Dimensions and Volumes in Transwell and MPS Devices**


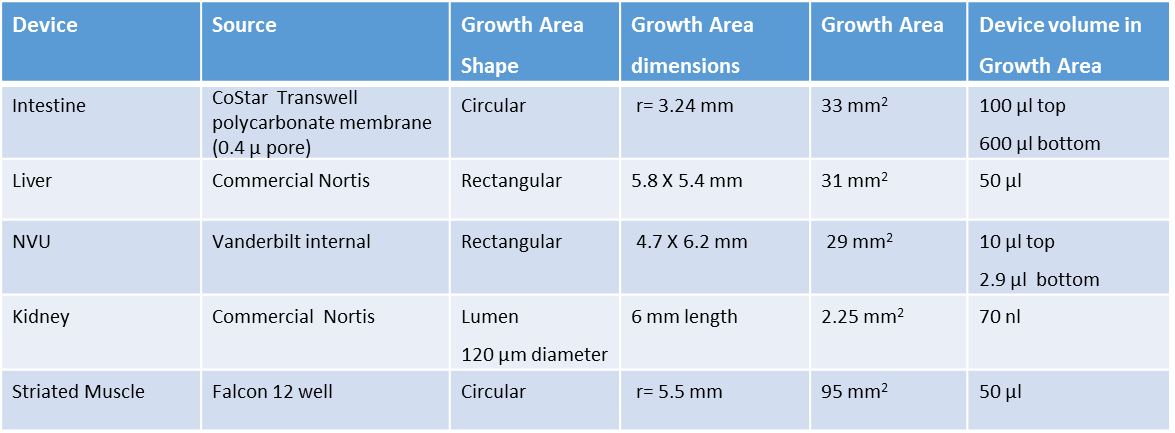


**Supplemental Table S8. TEER Test and 4 kD Fluorescent Dextran Polymer Diffusion in Human Intestine Model**

| **TEER Test (ohm cm^2^) 0 hr 24 hr** | **FTIC-Dextran Flux (% diffused at 24 hr) A → B^1^ B → A^2^** |
| --- | --- |
| 496 ± 78^3^ 650 ± 155^3^ | 0.1 ± 0.1%^3^ 0.4 ± 0.1%^3^ |

1. FITC-dextran added to apical chamber
2. FITC-dextran added to basolateral chamber
3. N = 4


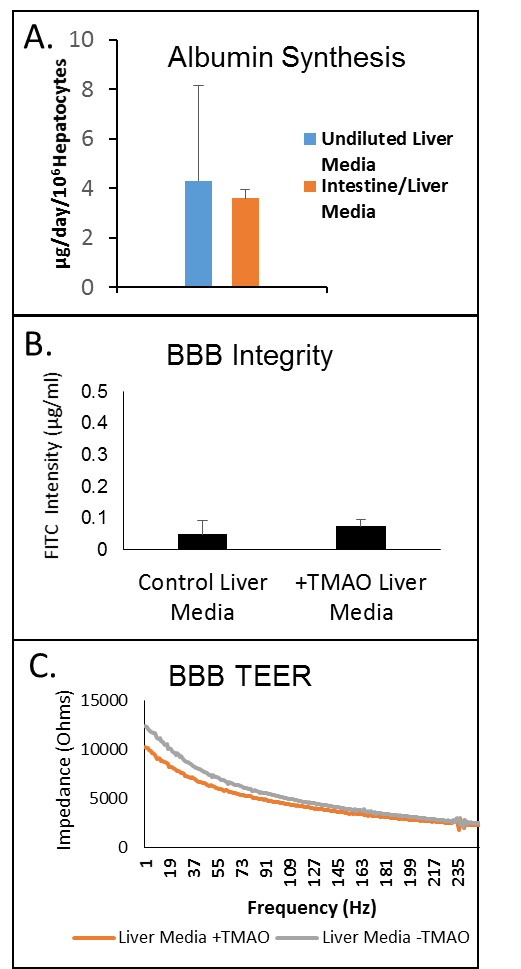


Supplemental Figure S1. Effect of mixed media on liver and NVU MPS functions. A) The mixture of intestinal conditioned media and liver media did not significantly impact synthesis of albumin in the liver model. B) 10 kD FITC dextran polymers did not penetrate the endothelial BBB in the NVU when treated to a mixture of liver conditioned media and EBM-2 media in the presence or absence of TMAO. C) TEER results in the NVU comparing liver media with and without TMA demonstrate intact endothelial BBB.

**Supplemental Figure S2. Recovery of 3 test compounds in 3 tubing materials and 2 biomaterials used to make devices.** (A) Level of recovery is best from polystyrene compared to PDMS. B) All test compounds were recovered from a 6-inch length polyetheretherketone (PEEK) tubing compared to the same length of modified block styrene-ethylene-butylene polymer (C-Flex) or bisphenol-1,2,3-triazol (Pharmed BPT).

## Additional details on the preparation of the SQL-SAL modules

The sequentially layered self-assembly liver module (SQL-SAL) for terfenadine and TMA experiments was constructed in a Nortis (Seattle, WA) microfluidic device as previously described(Fig. 1B)^60^. Dexamethasone, normally used to maintain hepatocyte differentiation, was removed from the liver media for functional coupling to avoid downstream toxicity to the human neurons in the NVU. For vitamin D3 experiments, the SQL-SAL model was constructed in 96-well microtiter plates to avoid the absorption of vitamin D3 and its metabolites by the PDMS in the Nortis device. Briefly, cryopreserved human hepatocytes were seeded into collagen type 1 coated 96-well plates at 26,000 hepatocytes/well and allowed to attach overnight in hepatocyte plating media (HPM). The HPM was decanted and 1.25x10^5^ Ea.Hy.926 endothelial cells and 8 X10^4^ differentiated U937 immune cells in MEM media supplemented with 10% FBS and L-Glutamine were added. After 2 h the media was decanted and 40 µL of a slurry of 1.5 mg/mL gelling collagen type 1 in Williams E media with 4 X 10^4^ LX-2 stellate cells was added. The collagen was allowed to gel for 1 h at 37^o^C, then 60 µL of Williams E Media supplemented with 1% FBS, 1.25 mg/mL BSA, 10 ug/ml insulin, 0.5 ug/ml transferrin and 7 ng/ml sodium selenite was added.

The 4-cell static SQL-SAL liver model was constructed in 96-well microtiter plates to test recovery and metabolism of vitamin D3 to 25-(OH) vitamin D3 in the presence and absence of DBP at molar ratios of 1:1 and 3:1 DPB:vitamin D3. Samples from the treatment conditions were collected after 24-h and 48-h incubations and stored in amber glass vials at -80°C for LC-MS/MS analysis.

***Additional details for functional coupling of the static liver with skeletal muscle for terfenadine toxicity testing***

For compound treatment, the static liver model was incubated with a dose of 10 µM terfenadine for 24 h. Media dosed with fresh terfenadine was changed daily. Liver function was assessed by measuring CYP3A4 activity using luminescence (ProMega), and toxicity assessed by lactate dehydrogenase (LDH) levels in the liver media, normalized to LDH levels produced by lysis buffer (ThermoFisher). The CYP3A4 activity following treatment with 10 µM terfenadine for 25 days was well fit by the following relationship: fraction of CYP3A4 activity = -0.463 ln(t, days)+1.4458. After 4 days’ exposure to 10 µM terfenadine, the estimated CYP3A4 activity as a fraction of initial activity was 0.80±0.25.

For treatment, myobundles were switched to media conditioned in the static liver model with or without terfenadine. LDH levels were measured in the skeletal muscle media and normalized to LDH levels produced by exposure to lysis buffer (ThermoFisher), to assess cell death. From 2 experiments, cell death based on LDH levels was 10.3±0.4% (mean±SD) after treatment of bundles with DMSO for 24 h and 23.2±8.2% after incubation with 10 µM terfenadine for 24 h. In one experiment, similar levels of cell death were observed when the myobundles were incubated for 24 h with the hepatocyte conditioned media DMSO (14%), hepatocyte conditioned media which metabolized terfenadine (8.3%) and hepatocyte conditioned media to which was added 10 µM terfenadine before incubation with myobundles (17.5%).

Myobundles were loaded into a custom-made force measurement apparatus containing a sensitive optical force transducer and a computer-controlled linear actuator (ThorLabs), as previously described^25^ Samples were stimulated (40 V/cm) to recreate twitch (1 Hz for 10 ms) and tetanic (20 Hz for 1 s) contraction. Contractile force traces were analyzed for peak twitch and tetanus force using a custom MATLAB program^2^. From experiments with three different donors, incubation of myobundles with 10 µM terfenadine for 24 h reduced the twitch force by 96.5±4.4% and the tetanus force by 95.4±1.9% (mean±SD).
